# Paired PET‐MRI Deep Learning Model for Translating [^11^C]PiB to [^18^F]Florbetaben Amyloid Images

**DOI:** 10.1002/mp.70168

**Published:** 2025-11-27

**Authors:** Cheng‐Han Tsai, Shao‐Yi Huang, Yu‐Nong Lin, Han‐Wei Wang, Ing‐Tsung Hsiao, Kevin T. Chen

**Affiliations:** ^1^ Department of Biomedical Engineering National Taiwan University Taipei Taiwan; ^2^ Department of Medical Imaging and Radiological Sciences Chang Gung University Taoyuan Taiwan

**Keywords:** amyloid PET, deformable convolution network, medical image translation

## Abstract

**Background:**

Amyloid PET imaging has been extensively employed in the noninvasive assessment of amyloid‐beta accumulation in Alzheimer's disease. Various amyloid radiotracers are commonly used in clinical settings; however, the limited interchangeability among these radiotracers hinders the feasibility of long‐term clinical trials and multicenter comparisons. The Centiloid method was proposed for standardization, though providing a single score per image; voxel‐wise translation remains a formidable task.

**Purpose:**

This paper proposes a U‐Net model based on a deformable convolution network (DCNv3‐based U‐Net) for [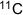]‐Pittsburgh compound B‐to‐[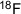]‐florbetaben image translation to augment existing datasets for large‐scale model training and provide image information when inconsistencies between visual assessments and the Centiloid scale occur.

**Methods:**

The DCNv3‐based U‐Net combined the benefits of deformable convolution that captures long‐range dependencies with efficient computation and the encoder–decoder architecture with skip connections for local–global feature learning and image synthesis.

**Results:**

The prediction images presented increased homogeneity to other previous models, closely resembling the texture of [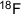]‐florbetaben.

**Conclusions:**

The DCNv3‐based U‐Net demonstrated high performance in metrics measurement and statistical analyses for the PET image‐to‐image translation task. This work also justified the importance of MR images in providing structural information.

## 1 INTRODUCTION

Alzheimer's disease (AD) is a neurodegenerative disease characterized by loss of memory, loss of cognitive function, and functional impairment, with associated neuropsychological symptoms. AD makes up 60%--80% of all dementia cases and is the fifth leading cause of death in adults older than 65 years. The total costs for the treatment of AD are estimated at $ 305 billion and are still growing substantially.^1^ The pathogenesis of AD remains unclear, with tau‐containing neurofibrillary tangles and amyloid‐beta plaques regarded as the primary proteins involved in the pathogenesis of AD.^2^ Amyloid‐beta radiotracers are essential tools in AD diagnosis per the National Institute on Aging–Alzheimer's Association (NIA‐AA) framework;^3^, ^4^ common radiotracers to image the amyloid‐beta biomarker include [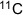]‐PiB (PiB)^5^ and the FDA‐approved [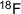]‐florbetaben (FBB),^6^ [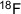]‐florbetapir,^7^ and [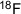]‐flutemetamol.^8^ With the continued development and approval of amyloid‐beta‐clearing drugs,^9^, ^10^, ^11^ the volume of amyloid‐beta PET imaging exams is expected to increase.^12^

To standardize the images acquired via the various amyloid‐beta radiotracers, amyloid‐beta calibration tasks can be executed by linear scaling methods (the Centiloid method).^13^ The Centiloid is a standardization method for quantitative amyloid imaging by linearly scaling the outcome of each particular amyloid radiotracer image on a 0 to 100 scale, anchored by young controls (0‐anchors, or YC‐0) and typical Alzheimer's disease patients (100‐anchors, or AD‐100). Other radiotracers can be converted to the Centiloid scale (also called CL) through a paired dataset, including the surrogate radiotracer and PiB scans, which are collected and calculated at the calibrating site. The calibration of the surrogate radiotracer with respect to the standard PiB is done through the calculation of standardized uptake value ratios (SUVr) using the standard cortex volumes of interest (VOI) on the Montreal Neurological Institute (MNI)‐152 space^14^ provided by the Centiloid Project (https://www.gaain.org/centiloid‐project). However, the Centiloid method's single value per image may lead to discordance between the visual assessment of specific brain regions and Centiloid quantification.

With the development of deep‐learning algorithms and the increase in computational power, deep‐learning applications in medical imaging such as the synthesis of full‐dose PET and CT images from their low‐dose counterparts and MR‐to‐CT translation^15^, ^16^ are numerous. In particular, PET studies regarding inter‐tracer image‐to‐image translation were also proposed, including the translation of SUVr and $K_{i}$ ratio [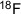]‐fludeoxyglucose (FDG) images to SUVr and distribution volume ratio [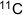]‐UCB‐J images.^17^ Generative artificial intelligence methods for image synthesis have also been proposed to address the calibration between different medical images, including [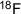]‐flortaucipir to FDG image‐to‐image translation,^18^ and a CycleGAN‐based method implemented to break through obstacles of interchangeability between two amyloid‐beta radiotracer images, translating PiB to [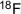]‐florbetapir and vice versa.^19^ Furthermore, a recent study also proposed a deep‐learning model (Swin UNetR) to generate FDG PET images from early‐phase [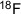]‐florbetapir and [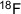]‐flutemetamol PET images,^20^ showing the feasibility of inter‐tracer PET image translation.

As there are numerous amyloid radiotracers available clinically but also requiring translation between the different radiotracer image pairs, herein, we proposed a deformable convolution‐based (DCNv3) U‐Net model for PiB‐to‐FBB image translation, leveraging the use of openly available paired datasets.

## 2 MATERIALS AND METHODS

### 2.1 Data collection

The public dataset provided by Rowe *et al.* was used in this study.^21^ The dataset contained 35 triplets of T1‐weighted MRI scans, PiB‐PET scans 50–70 min post‐injection, and FBB‐PET scans 90–110 min post‐injection, including 10 young, healthy controls (33±8 years), 6 elderly healthy controls (71.3±8 years), 9 patients with mild cognitive impairment (72±5 years), 8 patients with Alzheimer's disease (69±6 years), and 2 patients with frontotemporal dementia (74±8 years). Six subjects were acquired on a Philips Allegro PET camera in 3D mode and processed with rotating Cs‐137 point source attenuation correction; the other 29 subjects were acquired on a Philips TF64 PET/CT scanner with CT attenuation correction. Table 1 lists the demographics of the cohort. All subjects in our dataset were scanned under highly standardized and protocol‐driven conditions (i.e., 555 ± 10% MBq for PiB and 300 ± 10% MBq for FBB, with uptake windows consistent within the accepted ranges for each radiotracer). The Global Alzheimer's Association Interactive Networks (GAAIN) website provides more detailed information about this dataset (https://www.gaain.org/centiloid‐project).

**TABLE 1:** Demographics of all 35 subjects in the cohort provided by Rowe et al.^21^

Group	Number of subjects	Age (years)	MMSE score
Young healthy control (YHC)	10	33±8	>28
Elderly healthy control (EHC)	6	71.3±8	29±1
Mild cognitive impairment (MCI)	9	72±5	28±2
Alzheimer's disease (AD)	8	69±6	23±3
Frontotemporal dementia (FTD)	2	74±8	23±1

### 2.2 Data preprocessing

All image preprocessing steps were performed using PMOD 4.2 software (RRID: SCR_016547). Each subject's images were individually coregistered with their corresponding MR images. Next, skull stripping was performed on the MR images by using the segmentation function in PMOD. Subsequently, MR images were spatially normalized to the Chang Gung Memorial Hospital (CGMH) template space, a modified version of the automated anatomical labeling (AAL) atlas^22^ with a voxel size of $2\times 2\times 2\;{\mathrm{mm}}^{3}$. The SUVr, using the whole cerebellum as the reference region, was applied instead of the standard uptake value (SUV) in subsequent network training and analyses to unify the scale of the PET images.

**FIGURE 1:**
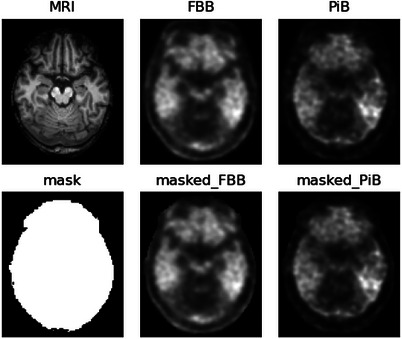
A representative subject showing the network inputs and the MRI‐based mask. MRI, medical resonance imaging.

The model was trained with 2D patient PiB (“PiB‐only”), and optionally MRI (“PiB+MR”) to investigate the value of MRI information in this image translation task, image slices (128×128 pixels) extracted from the original 3D volumes due to the limited cohort size, with the target FBB images as ground truth. Augmentations, including flipping, shifting, scaling, rotating, blurring, and the addition of noise, were applied to the input and the label in tandem to augment the size of the dataset for network training. Slices numbered between 10 and 75 in the CGMH template space were included for model training to limit the interference of blank and blurred images during training. Each subject's image was utilized as a mask to remove noise outside the brain region from the corresponding PET images; Zero padding was implemented on 2D images to augment the image size from (109, 91) to (128, 128) to match the input dimensions of the model. The MRI‐based head masks were then produced by morphological transformations and multiplied to the [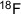]‐FBB PET and [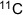]‐PiB PET. The examples of the mask and the images before and after masking are demonstrated in Figure 1.

### 2.3 Network structure

The proposed model, DCNv3‐based U‐Net, was inspired by the U‐Net and InternImage.^23^, ^24^ Previous studies have demonstrated the feasibility of medical image‐to‐image translation tasks using the U‐Net.^16^, ^17^ Recently, the InternImage network structure, an instance of the deformable convolution network (DCNv3), has been state‐of‐the‐art in object detection and semantic segmentation. The deformable convolution is a modified convolution operation in which the kernel employs adaptive offsets instead of a square grid in convolution, combining the benefits of adaptive spatial aggregation and efficient computation from convolutions with the advantage of capturing long‐range dependencies from the multi‐head self‐attention (MHSA)^25^ while achieving over 90% reduction in parameters compared with other MHSA‐based Nets such as SwinU‐Net and TransU‐Net (approximately 2M vs. 27M and 66M, respectively).

**FIGURE 2:**
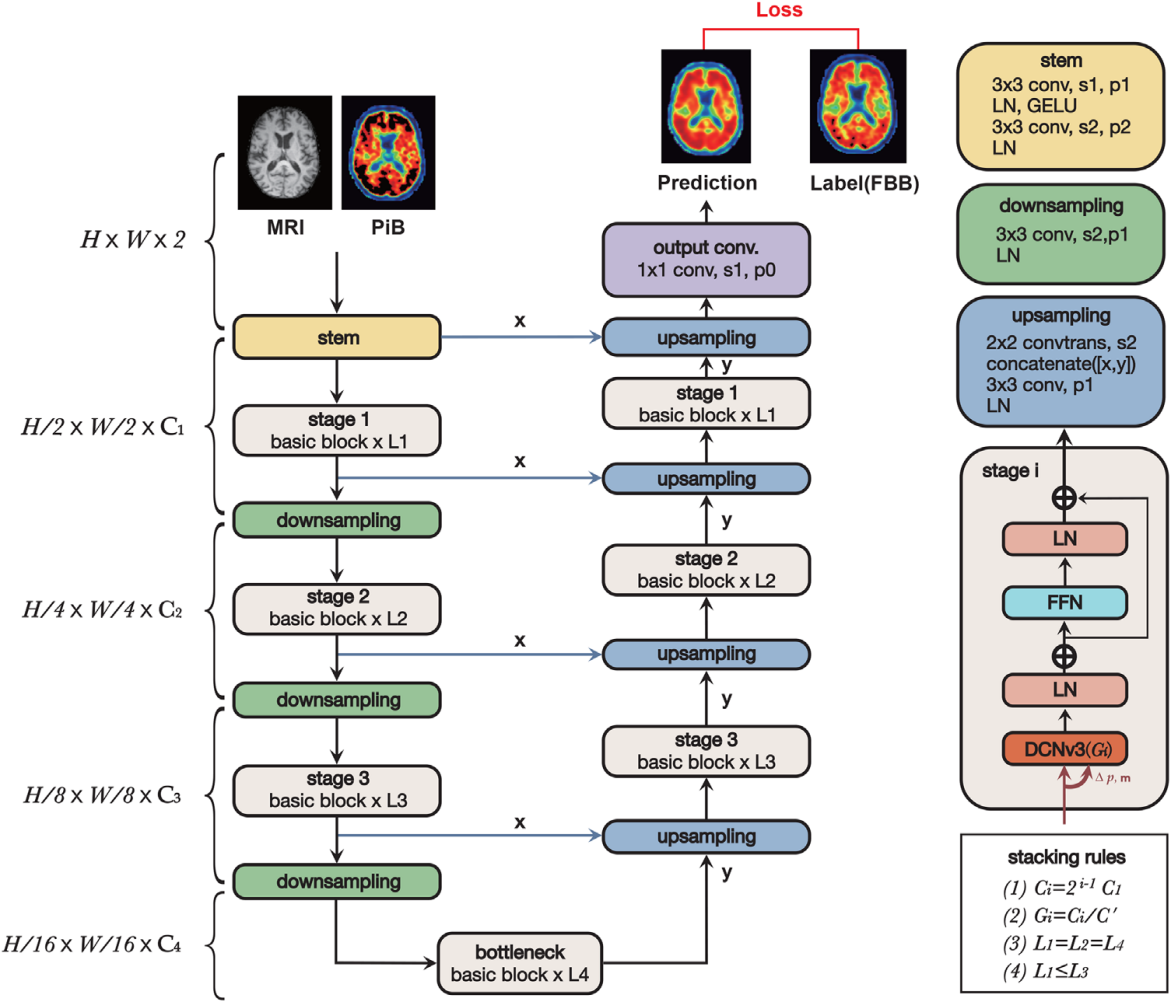
Architecture of the DCNv3‐based U‐Net.^26^ Constrained by the stacking rules, 4 hyperparameters $\left(C_{1},C^{\prime },L_{1},L_{3}\right)$ decide a model variant.^24^ $\left(C_{1},C^{\prime },L_{1},L_{3}\right)$ were set as (4,4,4,8) in our study. $C_{i}$, channel size in stage i; $L_{i}$, layer size in stage i; s, number of strides; *p*, number of paddings; LN, layer normalization; GELU, Gaussian error linear units function; convtrans, 2D transposed convolution; FFN, feed‐forward network; DCNv3, deformable convolutional network.

The model architecture is shown in Figure 2, where the double convolution layer in the U‐Net was replaced with the DCNv3 block. Considering the smaller image dimensions of medical images than natural images used in the original study, we modified the first convolution in the stem layer to a stride of one and padding of one to acquire similar feature maps as the previous study.^24^ The fourth basic block was regarded as the bottleneck to ensure the feature was compressed and extracted to the next layer. The upsampling layer contains a transposed convolution operation with a 2×2 kernel and a stride of two, a concatenating operation to combine the output of the last layer and the output from the skip connection, a convolution layer with padding of one, and a layer normalization layer. The output convolution layer was built by a single 1×1 convolution with a stride of one and zero padding to convert the channel and image sizes to the original.

### 2.4 Network training

The loss function was an L1‐L2‐structural similarity (SSIM^27^) mixed loss,^28^ defined as:

(1) $$L=\alpha 1*L_{1}\mathrm{loss}+\alpha 2*L_{2}\mathrm{loss}+\alpha 3*\left(1-\mathrm{SSIM}\right)$$

where α1, α2, and α3 are hyperparameters and set as α1=1, α2=0, and α3=1 according to our experimental result. The experimental details are provided in the Supplementary Information (Table S1).

To achieve better data utilization and training results, a five‐fold cross‐validation was applied by setting aside a fold for testing and a fold for validation during each training phase; the dataset was stratified by diagnosis and split into five equal folds. Since the hallmarks of FTD do not include amyloid‐beta deposition,^29^ we regarded FTD data as EHC to decrease the complexity of model training. In each step (fold), one of the groups was set as testing data (7 subjects), while the others were regarded as training and validation data (28 subjects). Subsequent analyses were conducted over all folds.

### 2.5 Data analysis

Metrics commonly used in the computer vision field, peak noise‐to‐signal ratio (PSNR), SSIM, and root‐mean‐square error (RMSE) were used to evaluate the image quality; the PET‐relevant SUV and SUVr were used to assess the accuracy of the predictions. Wilcoxon signed‐rank test^30^ compared the metric differences between image types due to the limited dataset.

Three visualization methods were applied: the relative change map, created by calculating pixel‐wise SUVr percentage differences; the Bland–Altman (BA) plot,^31^ and the correlation plot. These methods allowed for an understanding of what information the model learned or failed to learn, observation of noticeable relationships between the difference and the average, indicating the bias of the prediction, and confirmation that the prediction and ground truth were nearly identical. VOIs were selected from the CGMH template relevant to AD progression, including the global cerebral cortex, frontal cortex, cingulate cortex, lateral temporal cortex, medial temporal cortex, and superior parietal cortex, as well as the choroid plexus, where the uptake of PiB versus FBB is expected to be different, to demonstrate the performance of the proposed model. The Centiloid scale was also applied to compare across the [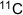]‐PiB‐derived CL values and those derived from FBB and our predictions. The standard VOIs provided by the Centiloid project were used.^13^, ^21^

## 3 RESULTS

### 3.1 Metrics

All predictions and original [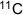]‐PiB PET images were compared with the ground truth ([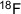]‐FBB) PET images by the metrics mentioned in Section 2.5. The PiB+MR prediction performed significantly better than the input PiB images, while the PiB‐only prediction had no significant difference from the control. As shown in Table 2, compared to the control (original PiB images vs. original FBB images), the metrics significantly improved when using PiB and MR images as inputs. Compared to previous models, our model also reported significantly better results in RMSE and SSIM (p<0.05).

**TABLE 2:** Comparison between models.

Configurations	RMSE ↓	PSNR ↑	SSIM ↑
Control	0.179±0.053	25.823±1.707	0.867±0.033
U‐Net	0.203±0.043	23.114±1.907	0.754±0.058
U‐Net 3+	$0.140\pm 0.030^{***}$	26.261±1.573	$0.907\pm 0.022^{***}$
TransU‐Net	0.579±0.317	$33.603\pm 6.631^{***}$	0.835±0.132
SwinU‐Net	$0.142\pm 0.040^{***}$	26.194±1.955	$0.898\pm 0.023^{***}$
PiB‐only	0.176±0.038	24.137±1.663	0.843±0.038
PiB+MR	$0.130\pm 0.032^{***}$	$26.848\pm 1.721^{***}$	$0.913\pm 0.020^{***}$

*Note*: The control group indicates the evaluation of the original PiB‐FBB pair. “*,” “**,” and “***” denote p<0.05, 0.01, 0.001, respectively. Note that U‐Net and TransU‐Net used L1 loss instead of the mixed L1‐L2‐SSIM loss, as they could not be trained stably with our original loss.

Abbreviations: PSNR, peak noise‐to‐signal ratio, RMSE, root‐mean‐square error, SSIM, structural similarity index measure.

### 3.2 Visual assessment

**FIGURE 3:**
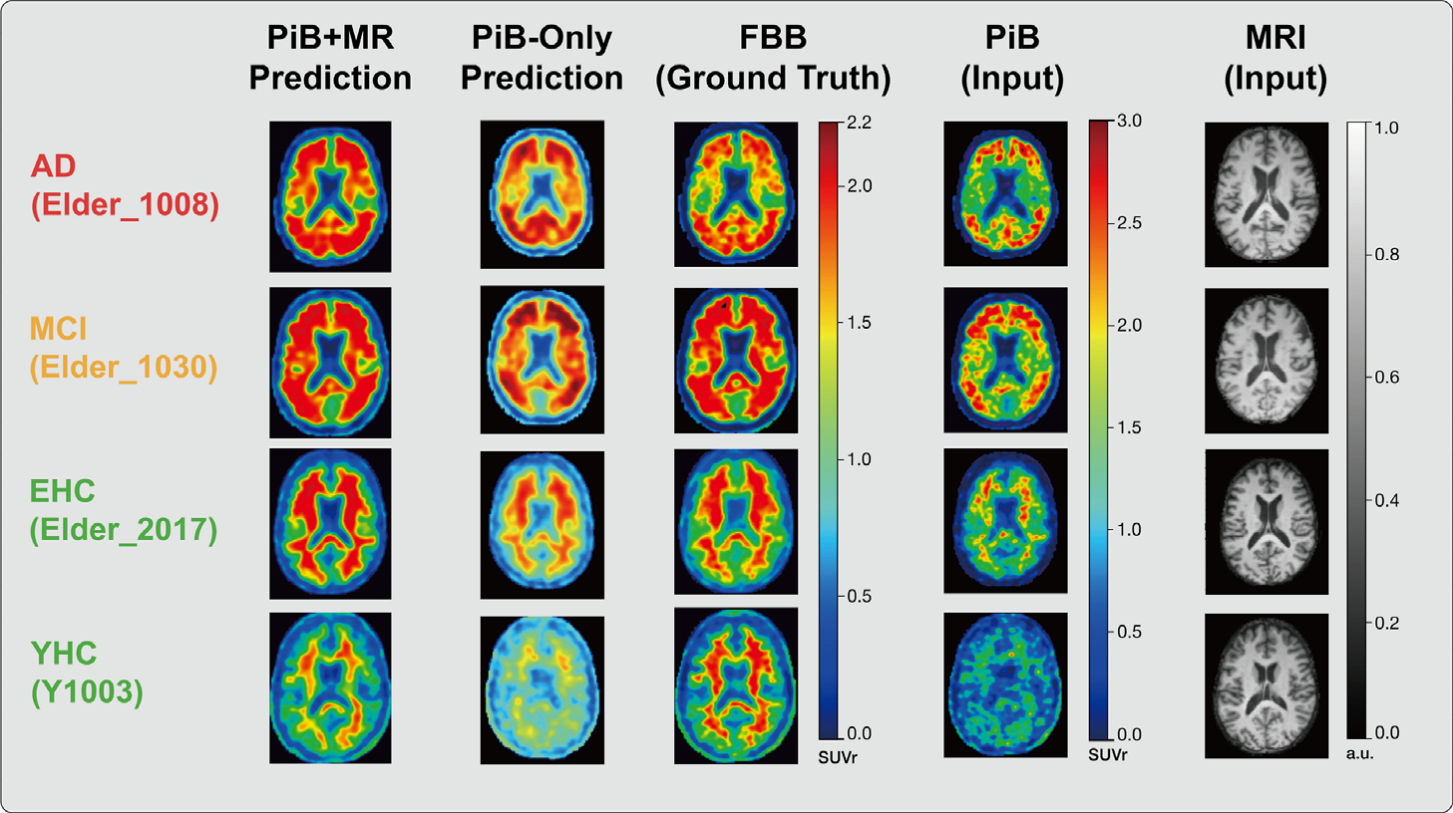
Visual assessment of representative participants. “PiB+MR Prediction” and “PiB‐Only Prediction” denote the data inputs used for training the DCNv3‐based U‐Net. AD, Alzheimer's disease; MCI, mild cognitive impairment; EHC, elderly healthy control; YHC, Young healthy control.

Figure 3 presented the images of 4 representative subjects from each diagnosis group for five image types. PiB images exhibited a wider range of SUVrs (approx. 0.0–3.0), compared to FBB images (approx. 0.0–2.2). Similar to FBB, non‐specific binding in white matter was observed in the predictions for EHC and YHC; cortical and subcortical gray‐matter binding was also present in the AD and MCI predictions. Images generated from the PiB+MR model, having acquired structural information from MRI, better reflected the underlying anatomical structures (such as the boundary between gray and white matter), especially for healthy controls and the amyloid‐negative cases, compared to those generated from the PiB‐only and other models.

When comparing across diagnoses, both configurations of the model predictions overestimated voxel values in the cases of AD and MCI, while underestimating the values in the cases of healthy controls in general. In the cases of EHC and YHC, the structure of the predictions was similar to that of MR images, with less information being derived from the input PiB, leading to lower similarity of PiB‐only predictions than the PiB+MR predictions due to the lack of MR image input.

### 3.3 Quantitative analyses

**FIGURE 4:**
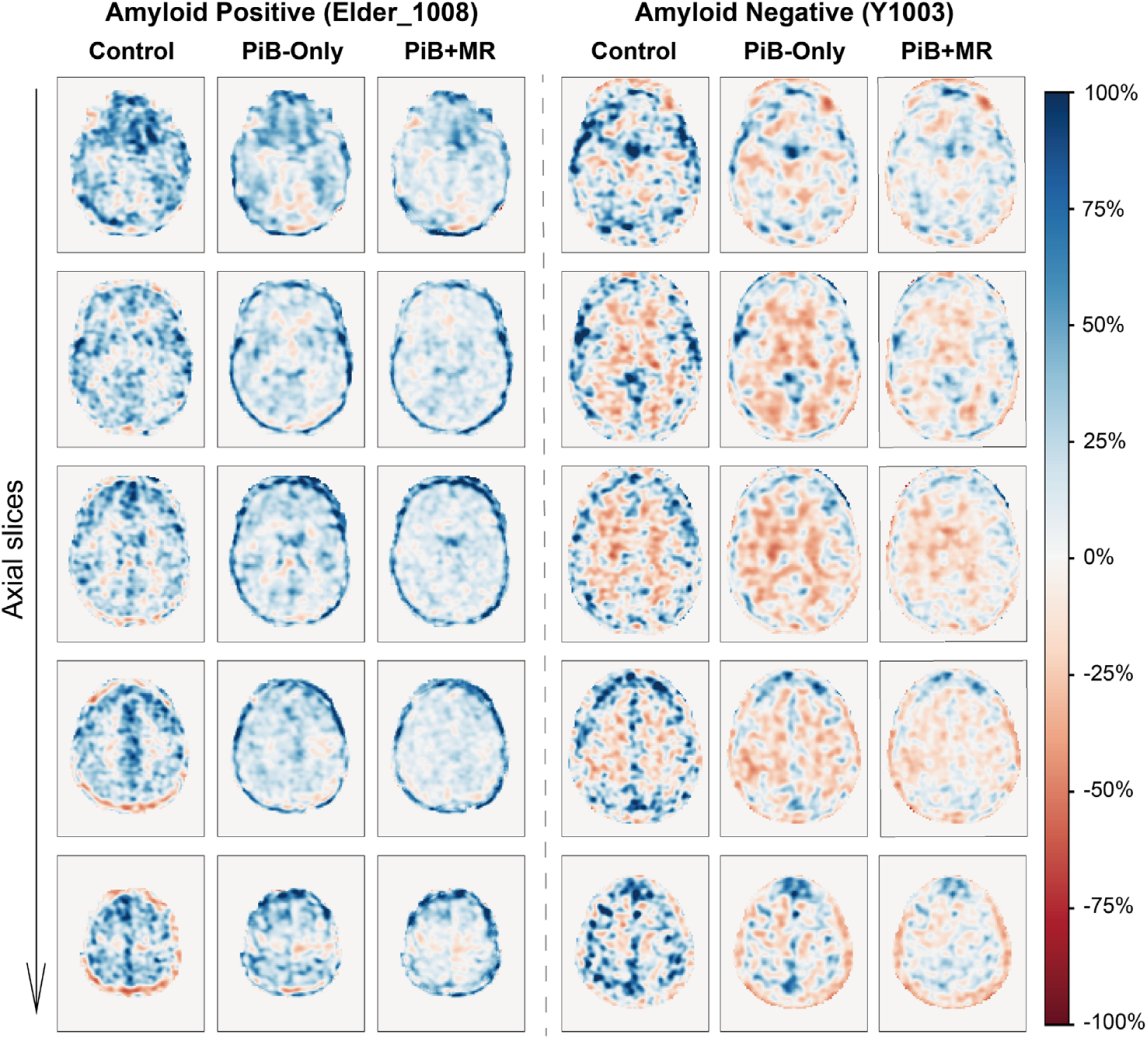
Relative change maps (five axial views numbered 25, 35, 45, 55, 65, from top to bottom) of representative amyloid‐positive and amyloid‐negative participants.

The relative change maps (RCM) of representative amyloid positive and negative subjects were shown in the axial view (Figure 4), where our model predictions showed reduced relative changes to the controls in general. The maps, as calculated between the predictions and the ground truth, were also generally smooth, suggesting a match in image texture between the two image types. However, for pixels near the skull, though not considered as a VOI for this population, the model overestimated and underestimated the voxel values for amyloid‐positive and amyloid‐negative individuals, respectively. When comparing the PiB‐only and PiB+MR predictions, the PiB‐only prediction exhibited larger discrepancies from the ground truth, particularly evident in certain anatomical structures, such as the ventricles in amyloid‐positive cases and the white matter in amyloid‐negative cases. The Supporting Information (Table S4) provided the RCs per subject. The results show that most predictions by the PiB+MR model had less RC than the other image types. Beyond RCM, the region‐wise mean SUVr values are shown in Table 3, indicating that the PiB+MR model exhibited a smaller absolute percentage difference from the ground truth than PiB‐only model in the whole brain, while comparable to the PiB‐only model in the cortical regions. The region‐wise SUVr values by subject are provided in the Supporting Information (Tables S2 and S3).

**TABLE 3:** Summary of the mean SUVr in representative regions.

Region	PiB‐only	PiB+MR
WB	−0.0406±0.0740	−0.0223±0.0677
Frontal	−0.004±0.0926	−0.0303±0.0807
Parietal	−0.0182±0.0884	−0.0295±0.0751
Temporal	−0.0104±0.0831	−0.0036±0.0570
Occipital	−0.0442±0.0643	−0.0426±0.0603
CP	−0.0250±0.0850	−0.0167±0.1033

Abbreviations: CP, choroid plexus; WB, whole brain.

**FIGURE 5:**
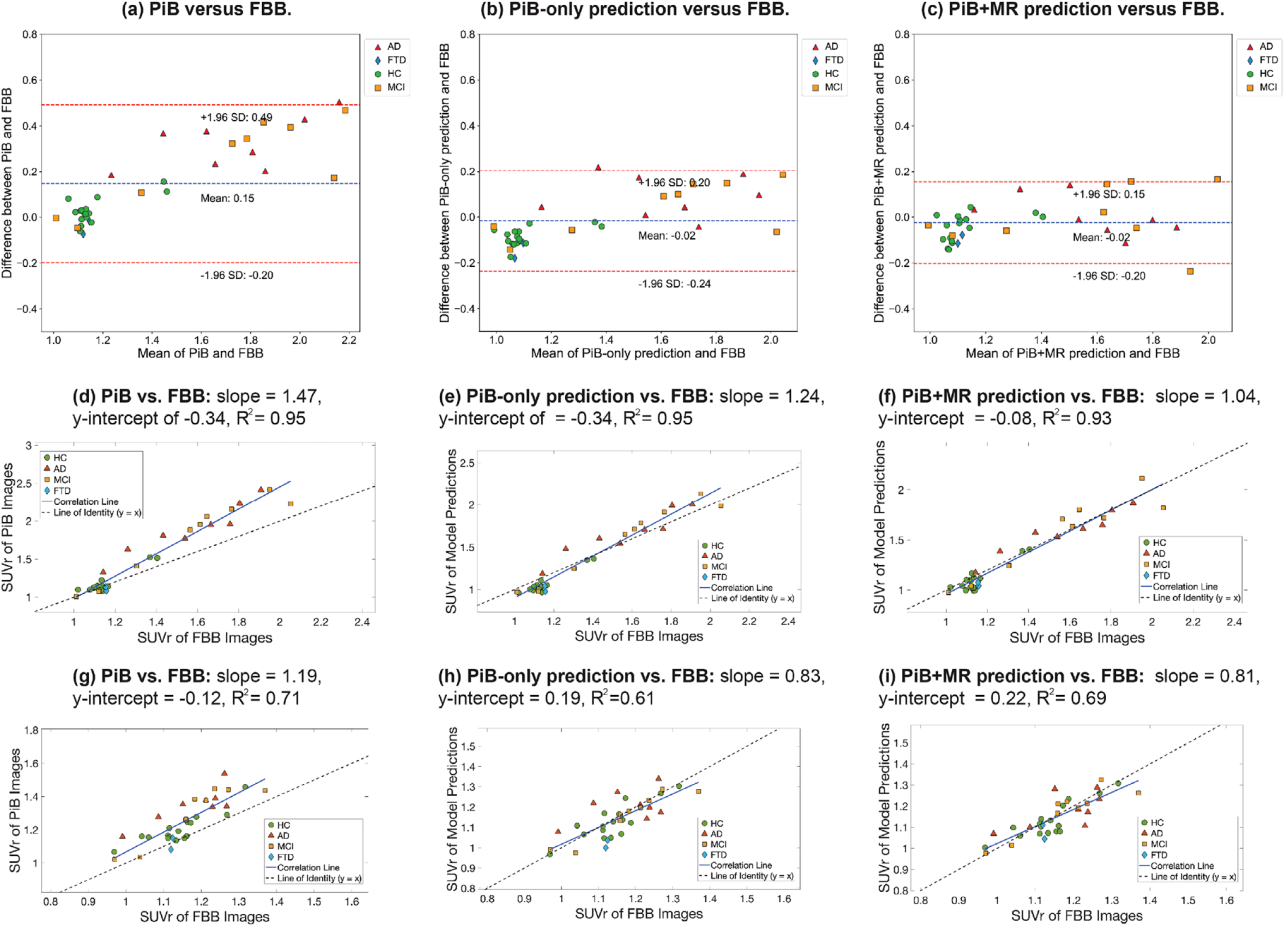
(a–c) BA plots of the mean SUVr in the cerebral cortex, comparing [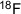]‐FBB images with the model predictions. (d–f) Correlation plots of the mean SUVr in the cerebral cortex. (g–i) Correlation plots of the mean SUVr in the medial temporal lobe. In the BA plots, the blue line represents the mean difference; the two red lines represent the upper and lower bounds of the 95% confidence interval. In the correlation plots, the dashed line and the blue line indicate the line of identity (y=x) and the correlation line, respectively. AD, Alzheimer's disease; FTD, frontotemporal dementia; HC, Healthy Control; MCI, mild cognitive impairment.

The Bland–Altman (BA) plot of the cerebral cortex is shown in Figure 5. The BA plot comparing PiB and FBB displayed a noticeable upward trend, indicating that the difference increased as the mean SUVr rose. In contrast, the BA plots comparing the model outputs that used either input versus FBB were more uniform. Additionally, regardless of input type, the model outputs achieved a mean closer to zero and demonstrated a narrower confidence interval than PiB.

The correlation plots (Figure 5) illustrate the changes observed before and after training. The correlation line for the PiB+MR predictions and FBB closely aligns with the line of identity (x=y, dashed line), indicating that the mean SUVr values from the two imaging types are nearly identical. Furthermore, the distribution of points remains consistent, suggesting that the model effectively preserves the data distribution while enhancing prediction accuracy. For comparison, the PiB‐only predictions resulted in a slope of 1.24, a y‐intercept of 0.34, and an $R^{2}$ of 0.95, whereas the PiB+MR predictions achieved a slope of 1.04, a *y*‐intercept of −0.08, and an $R^{2}$ of 0.93. These findings demonstrate that the PiB+MR predictions more accurately align with the true values than the PiB‐only predictions.

The equation of SUVr radiotracer to CL showed that the Centiloid method could be applied to our predictions accurately. The conversion equations of the PiB‐only and PiB+MR predictions had a higher slope and a lower intercept compared to those of the FBB. In particular, the CL derived from model predictions was strongly correlated with that of the FBB in the Wilcoxon test (0.94 for PiB‐only and 0.97 for PiB+MR).^13^ The CL regression equation and values across diagnostic groups are listed in Tables 4 and 5, respectively. The results showed that only one subject (elder_1015) was inconsistent when comparing the model‐predicted CL values with the ground truth.

**TABLE 4:** Centiloid conversion results.

	Correlation eq.	CL eq.
PiB‐only	$0.83x+0.08,R^{2}=0.99$	136.05x−141.06
PiB+MR	$0.72x+0.26,R^{2}=0.96$	135.36x−140.09
FBB (ground truth)	$0.61x+0.39,R^{2}=0.96$	153.4x−154.9

*Note*: The results demonstrated that the equations and the correlation coefficient were consistent between the ground‐truth FBB and the predictions when correlating to PiB.

Abbreviation: FBB, [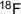]‐florbetaben.

**TABLE 5:** Centiloid values across diagnostic groups.

ID	Diagnostic	CL_FBB	CL_PiB_only	CL_PiB_MR	CL_PiB
elder_1008	AD	39.14	65.78	58.97	64.14
elder_1009	AD	92.67	99.9	87.89	93.81
elder_1015	AD	15.78	26.9	24.12	30.53
elder_1023	AD	63.75	60.86	63.75	65.01
elder_1028	AD	118.43	108.22	105.02	118.27
elder_1031	AD	87.72	70.03	70.62	71.14
elder_1032	AD	75.97	69.67	67.97	72.25
elder_1034	AD	44.18	62.22	66.29	62.45
elder_1019	FTD	10.58	2.88	5.18	1.2
elder_2029	FTD	10.8	7.13	8.03	7.59
elder_1010	HC	12.24	18.57	15.77	16.88
elder_2002	HC	4.37	5.62	19.26	5.98
elder_2005	HC	44.35	39.96	46.75	39.15
elder_2017	HC	1.39	8.29	8.3	7.58
elder_2030	HC	8.46	9.26	13.46	9.78
elder_2032	HC	36.08	40.53	48.12	40.71
Y1001	HC	9.14	8.57	10.5	7.43
Y1002	HC	7.85	5.01	−4.51	4.68
Y1003	HC	−6.48	−0.04	0.67	0.56
Y1004	HC	7.25	5.92	−0.95	8.06
Y1005	HC	13.47	8.54	7.1	7.84
Y1007	HC	15.88	3.57	1.1	4.43
Y1011	HC	13.66	9.19	2.78	11.23
Y1012	HC	14.02	9.54	3.75	8.68
Y1017	HC	11.17	14.33	16.61	13.27
Y1021	HC	13.07	12.54	17.97	11.49
elder_1018	MCI	6.33	1.79	2.86	1.84
elder_1022	MCI	123.37	97.35	90.37	91.43
elder_1024	MCI	2.6	6.78	2.31	5.05
elder_1026	MCI	40.9	36.54	34.04	39.06
elder_1029	MCI	75.03	80.13	88.59	81.42
elder_1030	MCI	62.98	65.25	64.1	66.56
elder_1036	MCI	88.43	93.65	82.62	90.46
elder_1037	MCI	119.29	121.33	133.44	117.31
elder_1038	MCI	60.2	68.24	81.18	66.77

Abbreviations: AD, Alzheimer's disease; FTD, frontotemporal dementia; HC, Healthy Control; MCI, mild cognitive impairment.

## 4 DISCUSSION

This study proposed the DCNv3‐based U‐Net trained by supervised learning on a paired dataset for voxel‐wise translation of PiB to FBB scans. As patients with cognitive disorders require long‐term follow‐up, as well as for clinical trials, an interchangeable quantification of amyloid deposits using different radiotracers is needed when patient cohorts include those who have had [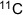]‐PiB, the first amyloid radiotracer, PET images while others (or even the same patient at a different time point) were scanned with other FDA‐approved radiotracers such as FBB. Although the Centiloid method provided an interchangeable and standardized quantification scale, it provided a single value for each subject. As the spatial pattern of amyloid deposits is associated with the progression of amyloid‐related diseases,^32^ misjudgment could occur using the Centiloid method without considering the spatial pattern of the amyloid burden. Moreover, subsets of dementia contain different amyloid distribution patterns.^33^, ^34^ Therefore, several advantages exist in providing regional SUVrs and image voxel‐level analyses. Our FBB‐like PiB images showed high accuracy in image conversion through the global and regional SUVr. The importance of MRI was observed in our study; the predictions acquired the structural information from the respective MR scans and generated FBB uptake when the PiB images lacked amyloid radiotracer activity.

The visual assessment demonstrated that the trained model generated smoother images than the ground truth due to the model's encoder–decoder structure, which extracted features from input images and generated predictions through up‐sampling, eliminating image noise in PET images. Our model applied the DCNv3 operation, which enhanced the ability of long‐range dependency and adaptive spatial aggregation; thus, the results of our model were more precise than those of the other models. The visual assessment also suggested that the model could generate accurate predictions when the PET images consisted of low‐uptake voxels, such as those in healthy subjects, where the model could acquire structural information from the input MRI. The relative change maps also revealed that the brain voxel predictions from our model were more similar to the ground truth than the input images.

The correlation plots' results suggest that regional and global cortical SUVr ground truth values were nearly identical (regardless of diagnoses) to our predictions, showing that the different disease populations did not bias our model training. Of all regions examined (data not shown), the lowest $R^{2}$ occurred in the medial temporal lobe (MTL) for two reasons: 1. MTL contains a relatively lower SUVr uptake than the cerebral cortex (0.9–1.4 and 1.0–2.2 in MTL and the cerebral cortex, respectively), which implies relatively low amyloid radiotracer activity in this region, providing insufficient information on the PET imaging training models to predict the region. 2. The MTL is a small region, and errors from coregistration and data augmentation could affect the performance of the model training dramatically. Compared with previous studies,^20^ our correlation performance (slope=1.04, $R^{2}=0.93$) is comparable to the reported values (slope = 0.93–1.00, $R^{2}=$ 0.68–0.72). While we did not assess additional measures such as CSS or Dice similarity, our regression results demonstrate that the proposed method preserves quantitative relationships between modalities at a level similar to prior work.

The BA plots additionally justify the consistency of our models. The BA plots comparing the PiB and the FBB show an increased bias with higher mean SUVrs, highlighting the differences in regional uptake patterns between the two radiotracers. By contrast, low bias and variance in BA plots between our model (regardless of inputs) and the FBB further suggest that the model predictions were able to accurately predict FBB images, regardless of the diagnoses or the regional uptake patterns of the original PiB. Moreover, compared to the Centiloid method, our work provided a full image to evaluate the amyloid accumulation instead of through a single value while retaining the ability to transfer to CL.

Using the commonly applied 20 CL cutoff for amyloid positivity,^35^ only one equivocal subject (elder_1015) was reclassified when comparing model‐predicted CL values with the ground truth, indicating that the model predictions are largely consistent with established clinical thresholds. However, further validation in larger cohorts is necessary to confirm the reliability of the model for clinical decision‐making. Additionally, no false negatives were observed; although one false positive occurred in our predictions, sensitivity is more crucial in the early detection of AD. The implementation is sufficiently reliable to assist doctors in identifying potential patients before a detailed reading of the scans.

The proposed model leveraged the ability of deformable convolution to adapt to the irregular ROIs in the brain. To our knowledge, MHSA could also be applied to image‐to‐image translation; however, its training process was more space‐expensive. The DCNv3‐based U‐Net achieved comparable performance to the MHSA‐based models (SwinU‐Net and TransU‐Net) while requiring fewer parameters.

There are several limitations to our work. The first challenge is obtaining a large cohort of individuals who have been scanned with multiple amyloid radiotracers. With a dataset limited in size yet diverse in individual diagnoses, we trained a DCNv3‐based U‐Net using supervised learning. Although it outperformed other models, a larger dataset would be necessary for training a more complex network with higher capacity. Another limitation is that the model implicitly assumes a fixed radiotracer dose. Although the dose used in our dataset reflects the commonly adopted clinical protocol, alternative dosing strategies are also used in practice. As a result, generalization to non‐standard doses remains to be validated. Furthermore, image translations in more than one direction are necessary to increase interchangeability or standardization among multiple amyloid PET radiotracers; however, training separate supervised models for each radiotracer pair is not an efficient approach. In addition, although the current model input did not include clinical information (e.g., age, diagnoses), incorporating such data could further improve performance. A reader study is also essential for clinical deployment, as it would provide evidence of the model's utility and acceptance in routine practice. Finally, the use of two‐dimensional input disregards the continuity between slices along the vertical axis, which could be addressed with a three‐dimensional architecture in future work.

## 5 CONCLUSION

In this work, we introduced the DCNv3‐based U‐Net, a novel model for the PET image‐to‐image translation task. Leveraging the advantages of the DCNv3 operator, the model could predict PET radiotracer uptake patterns more precisely than the previous models and more closely match the brain structure. Despite the limited size of the dataset, the model was well‐tuned and outperformed the other models. Additionally, the Centiloid method was applicable to the model predictions. With this model, a potential application of the proposed method is generating pseudo‐FBB images from previous PiB images for long‐term trials. Another application is providing modality translation and an image‐based alternative when inconsistencies between visual assessments and the Centiloid scale occur. Future works will include training the model with a larger dataset and examining the model's generalizability to different configurations and radiotracers.

## CONFLICT OF INTEREST STATEMENT

The authors have no conflicts to disclose.

## Supporting information

Supporting Information

## Data Availability

The data and code supporting the findings of this study are available at https://github.com/NTUMMIO/Amyloid_tracer_conversion.

## Appendix

[Boxed-text mp70168-fea-0001], A.1, A.2

ALGORITHM A.1 MR masking. **Table:**

**Input**: PiB‐PET, FBB‐PET, MRI
**Output**: PiB‐PET and FBB‐PET with MR‐mask
// Kernel initialization
kernel ←5×5 all‐ones matrix
**for all** Patient data in {MRI, PiB, FBB} **do**
// Thresholding MRI to create mask
mask ← threshold (input=MRI, range=[0.005,1], scalar_methods=MINMAXSCALAR)
// Mask erosion
mask ← erode (kernel=kernel, iteration=1)
// Mask dilation
mask ← dilate (kernel=kernel, iteration=2)
// Apply mask to PiB‐PET and FBB‐PET
masked‐PiB‐PET ← mask ∗ PiB‐PET
masked‐FBB‐PET ← mask ∗ FBB‐PET
**end for**
**return** masked‐PiB‐PET, masked‐FBB‐PET

**TABLE A.1:** Experimental configurations.

Parameters	Settings
Drop path rate	0.1
Depths	[4,4,8,4]
Groups	[2,4,6,8]
Channels	32
Hyperparameters of loss function	[$\alpha_{1},\alpha_{2},\alpha_{3}$] = [1,0,1]

*Note*: The loss function is formatted as $L=\alpha_{1}*L_{1}\mathrm{Loss}+\alpha_{2}*L_{2}\mathrm{Loss}+\alpha_{3}*\left(1-\mathrm{SSIM}\right)$.

**TABLE A.2:** Training configurations.

Configs	Settings
Batch size	8
Training epochs	100
Base learning rate (LR)	$10^{-4}$
Min LR	$5\times 10^{-6}$
LR scheduler	cosine annealing schedule
max iterations of scheduler	100 epochs
Early stop	10
