# Supplementary material for: Paired PET‐MRI Deep Learning Model for Translating [11C]PiB to [18F]Florbetaben Amyloid Images
Source: Med Phys. 2025 Nov 27;52(12):e70168. doi: 10.1002/mp.70168 (PMC12660554; doi:10.1002/mp.70168)
Supplement: Supplementary file 1 — Supporting Information [file MP-52-0-s001.pdf]

Table S.1: Ablation study: loss function hyperparameter $(\alpha_1, \alpha_2, \alpha_3)$ tuning. The equation was formulated as: $L = \alpha_1 \cdot \mathrm{L_1\ Loss} + \alpha_2 \cdot \mathrm{L_2\ Loss} + \alpha_3 \cdot (1 - \mathrm{SSIM})$ (Mean ± SD). *Params* in the table denote the value of hyperparameters $[\alpha_1, \alpha_2, \alpha_3]$.

Name	Params	RMSE ↓	PSNR ↑	SSIM ↑
Control	-	0.179 ± 0.053	25.823 ± 1.707	0.867 ± 0.033
Config 1	1,0,0	0.130 ± 0.034***	26.842 ± 1.759***	0.913 ± 0.020***
Config 2	0,1,0	0.142 ± 0.030***	25.938 ± 1.376	0.697 ± 0.026
Config 3	1,1,0	0.142 ± 0.030***	25.995 ± 1.393	0.707 ± 0.025
Config 4	1,1,0.5	0.129 ± 0.034***	26.877 ± 1.704***	0.911 ± 0.020***
Config 5	1,1,1	0.129 ± 0.032***	26.871 ± 1.660***	0.913 ± 0.020***
Config 6	1,0,1	0.130 ± 0.032***	26.848 ± 1.721***	0.913 ± 0.020***

Author Manuscript

This article is protected by copyright. All rights reserved.

Table S.2: Region-wise absolute bias of **PiB+MR** to FBB images. Abbreviations used: WB, whole brain; CP, choroid plexus.

ID	WB	frontal	parietal	temporal	occipital	CP
elder_1008	0.0929	0.1151	0.0592	0.0867	0.0614	0.0939
elder_1009	-0.0138	-0.0171	-0.0290	0.0076	-0.0844	-0.0432
elder_1010	-0.0339	-0.0429	-0.0070	0.0145	-0.0138	-0.0018
elder_1015	-0.0099	-0.0149	0.0798	0.0253	0.0336	-0.0618
elder_1018	-0.0840	-0.1069	-0.0960	-0.0549	-0.0670	-0.0693
elder_1019	-0.1163	-0.0938	-0.1039	-0.0496	-0.0254	-0.0114
elder_1022	-0.1179	-0.1576	-0.1318	-0.1151	-0.1412	-0.1562
elder_1023	-0.0118	-0.0106	-0.0138	0.0271	-0.0472	-0.0991
elder_1024	-0.0465	-0.0435	-0.0475	-0.0216	-0.0432	-0.0304
elder_1026	-0.0445	-0.0130	-0.0495	-0.0343	-0.0810	-0.0615
elder_1028	-0.0059	-0.0407	-0.0417	-0.0039	-0.0810	0.0143
elder_1029	0.0656	0.1022	0.1182	0.1218	0.0654	-0.0230
elder_1030	0.0031	0.0436	0.0416	0.0416	0.0116	-0.0252
elder_1031	-0.0712	-0.0539	-0.0696	-0.0229	-0.0737	-0.0783
elder_1032	-0.0369	-0.0689	-0.0391	-0.0588	-0.0555	-0.0271
elder_1034	0.1303	0.1485	0.0586	0.1352	0.0069	0.1348
elder_1036	-0.0142	-0.0123	-0.0558	-0.0275	-0.0688	0.0038
elder_1037	0.0789	0.0760	0.0845	0.0658	0.0065	0.3560
elder_1038	0.0875	0.1101	0.1157	0.0501	0.0767	0.2041
elder_2002	0.0708	0.0038	0.0185	0.0570	-0.0206	0.0459
elder_2005	0.0154	-0.0535	-0.0122	0.0086	-0.0208	-0.1294
elder_2017	0.0038	-0.0380	-0.0134	-0.0670	-0.0482	-0.0556
elder_2029	-0.0307	-0.0299	-0.1053	-0.0411	-0.1221	-0.1424

Author Manuscript

This article is protected by copyright. All rights reserved.

elder_2030	0.0134	-0.0398	-0.0569	-0.0184	-0.0796	0.0357
elder_2032	0.0473	0.0789	-0.0071	0.0028	-0.0315	-0.0468
Y1001	-0.0286	-0.0294	-0.0314	-0.0009	-0.0378	0.0609
Y1002	-0.1157	-0.1755	-0.1657	-0.0506	-0.1319	-0.0251
Y1003	-0.0322	0.0003	0.0282	0.0541	0.0428	-0.0456
Y1004	-0.1052	-0.1283	-0.1095	-0.0435	-0.0961	-0.0920
Y1005	-0.1034	-0.0748	-0.0923	-0.0331	-0.0731	-0.0269
Y1007	-0.1296	-0.1677	-0.1616	-0.0653	-0.1253	-0.1039
Y1011	-0.0794	-0.1361	-0.1301	-0.0768	-0.1413	-0.0525
Y1012	-0.1036	-0.1090	-0.0914	-0.0684	-0.0977	-0.1758
Y1017	0.0019	-0.0531	-0.0175	-0.0122	-0.0356	0.1044
Y1021	-0.0560	-0.0262	0.0417	0.0422	0.0465	-0.0532
**Mean**	-0.0223	-0.0303	-0.0295	-0.0036	-0.0426	-0.0167
**std**	0.0677	0.0807	0.0751	0.0570	0.0603	0.1033

Author Manuscript

This article is protected by copyright. All rights reserved.

Table S.3: Region-wise absolute bias of **PiB-only** to FBB images. Abbreviations used: WB, whole brain; CP, choroid plexus.

ID	WB	frontal	parietal	temporal	occipital	CP
elder_1008	0.1164	0.1965	0.1532	0.18	0.135	0.0646
elder_1009	0.0577	0.1328	0.102	0.1077	0.0191	0.0282
elder_1010	-0.0237	-0.0078	-0.0472	-0.0508	-0.0304	0.1748
elder_1015	0.0073	0.0353	0.0734	0.0461	0.0416	0.1049
elder_1018	-0.1218	-0.1403	-0.1586	-0.1442	-0.1363	-0.0144
elder_1019	-0.1713	-0.1226	-0.1282	-0.1269	-0.085	-0.0237
elder_1022	-0.077	-0.0506	-0.0363	-0.0201	-0.0773	-0.1638
elder_1023	-0.0175	0.0483	0.0024	0.0413	-0.0534	-0.0922
elder_1024	-0.0472	-0.0317	-0.0508	-0.0265	-0.0274	0.0465
elder_1026	-0.0492	0.0288	-0.0425	-0.05	-0.0991	-0.0119
elder_1028	0.0224	0.0533	0.0422	0.0559	-0.024	0.0555
elder_1029	0.0399	0.0937	0.1161	0.1145	0.0522	0.0101
elder_1030	0.0258	0.1373	0.1139	0.0731	0.0188	-0.0007
elder_1031	-0.046	0.0131	-0.023	0.0097	-0.0836	-0.085
elder_1032	-0.0045	0.0258	0.0387	0.0255	-0.0427	-0.0103
elder_1034	0.1124	0.1653	0.0877	0.1643	0.0076	0.1459
elder_1036	0.0534	0.1405	0.096	0.067	0.018	0.0822
elder_1037	0.0508	0.1047	0.1258	0.1037	0.0309	0.1361
elder_1038	0.0379	0.0933	0.088	0.0547	0.032	0.1107
elder_2002	-0.0583	-0.0871	-0.1085	-0.1001	-0.1326	0.0154
elder_2005	-0.045	-0.0508	-0.0159	0.0102	-0.0564	-0.0924
elder_2017	-0.0309	-0.0338	-0.063	-0.1073	-0.0716	0.0812
elder_2029	-0.0742	-0.0554	-0.1179	-0.0786	-0.1437	-0.1232

Author Manuscript

This article is protected by copyright. All rights reserved.

elder_2030	-0.0594	-0.0835	-0.1076	-0.0587	-0.1161	0.0616
elder_2032	-0.0124	0.0305	-0.0181	-0.0204	-0.0433	-0.0101
Y1001	-0.0811	-0.0664	-0.0715	-0.0664	-0.0723	0.1042
Y1002	-0.1085	-0.1002	-0.1073	-0.0585	-0.1197	0.1291
Y1003	-0.1039	-0.0004	0.0035	-0.0084	0.0028	0.0124
Y1004	-0.1432	-0.0725	-0.066	-0.0883	-0.069	-0.0444
Y1005	-0.1427	-0.064	-0.095	-0.0714	-0.0677	0.0089
Y1007	-0.1603	-0.1527	-0.1643	-0.1129	-0.1458	-0.0071
Y1011	-0.0828	-0.0843	-0.0975	-0.0887	-0.1068	0.0939
Y1012	-0.1192	-0.0915	-0.0724	-0.0565	-0.0522	-0.1021
Y1017	-0.0554	-0.0765	-0.0594	-0.0543	-0.0397	0.1712
Y1021	-0.1078	-0.0665	-0.0286	-0.0275	-0.0093	0.0181
**Mean**	-0.0406	-0.004	-0.0182	-0.0104	-0.0442	0.025
**std**	0.074	0.0926	0.0884	0.0831	0.0643	0.085

Author Manuscript

This article is protected by copyright. All rights reserved.

Table S.4: The absolute mean and standard deviation of voxel-wise SUVr relative change over an MR-derived head mask between translated PiB-only, PiB+MR, and ground-truth FBB images (Mean ± SD). The relative change was calculated as: $100 \times \left|SUVr_{translated} - SUVr_{FBB}\right| - SUVr_{FBB}$. Note that *Ref* denotes the input PiB SUVr.

Name	Ref Mean	Ref STD	PiB-only Mean	PiB-only STD	PiB+MR Mean	PiB+MR STD
elder_1008	27.05%	20.97%	24.32%	21.61%	20.15%	21.31%
elder_1009	26.78%	18.10%	18.26%	14.15%	15.73%	14.46%
elder_1010	15.90%	12.55%	17.08%	13.39%	15.40%	12.76%
elder_1015	19.41%	17.34%	14.80%	12.88%	14.14%	13.30%
elder_1018	19.57%	17.09%	20.97%	14.23%	17.18%	13.60%
elder_1019	23.89%	21.94%	19.42%	14.42%	16.48%	13.58%
elder_1022	21.33%	16.92%	17.85%	14.78%	20.16%	14.28%
elder_1023	19.69%	15.66%	13.89%	11.49%	11.82%	10.83%
elder_1024	15.98%	15.69%	15.12%	17.22%	18.11%	23.52%
elder_1026	18.39%	15.20%	15.03%	13.23%	13.99%	12.96%
elder_1028	23.57%	17.52%	13.43%	11.96%	11.26%	10.66%
elder_1029	25.57%	20.08%	21.70%	21.60%	29.14%	35.14%
elder_1030	23.96%	21.65%	20.73%	23.37%	18.12%	23.35%
elder_1031	17.71%	15.00%	13.32%	12.28%	13.71%	12.63%
elder_1032	19.44%	14.37%	13.41%	10.89%	12.76%	10.95%
elder_1034	25.78%	17.87%	18.25%	13.35%	16.71%	12.70%
elder_1036	22.61%	15.30%	14.68%	11.30%	12.16%	11.32%
elder_1037	29.39%	186.08%	18.63%	25.16%	21.16%	28.68%
elder_1038	21.02%	17.17%	14.42%	13.82%	20.77%	22.61%
elder_2002	16.76%	14.34%	15.63%	13.28%	18.77%	18.59%
elder_2005	17.33%	14.40%	12.70%	10.28%	12.17%	10.76%

Author Manuscript

This article is protected by copyright. All rights reserved.

elder_2017	16.59%	13.47%	14.70%	11.78%	13.68%	11.55%
elder_2029	19.06%	15.87%	17.11%	12.78%	15.50%	11.98%
elder_2030	15.63%	12.35%	17.59%	13.22%	14.10%	10.56%
elder_2032	18.33%	13.56%	15.03%	11.47%	13.97%	10.28%
Y1001	15.73%	15.99%	15.79%	15.35%	13.88%	15.73%
Y1002	22.22%	23.29%	18.25%	17.26%	17.83%	16.89%
Y1003	27.45%	25.45%	20.21%	16.56%	15.14%	13.13%
Y1004	20.25%	18.25%	17.55%	12.98%	15.74%	12.91%
Y1005	25.00%	26.70%	22.34%	21.28%	18.72%	18.60%
Y1007	17.92%	13.77%	20.43%	13.00%	18.64%	12.57%
Y1011	17.48%	14.36%	16.33%	12.30%	15.60%	11.71%
Y1012	20.38%	15.74%	17.54%	12.76%	15.45%	12.15%
Y1017	21.61%	21.37%	19.70%	19.87%	23.76%	27.97%
Y1021	18.71%	15.47%	17.96%	13.25%	16.67%	13.55%

Author Manuscript

This article is protected by copyright. All rights reserved.

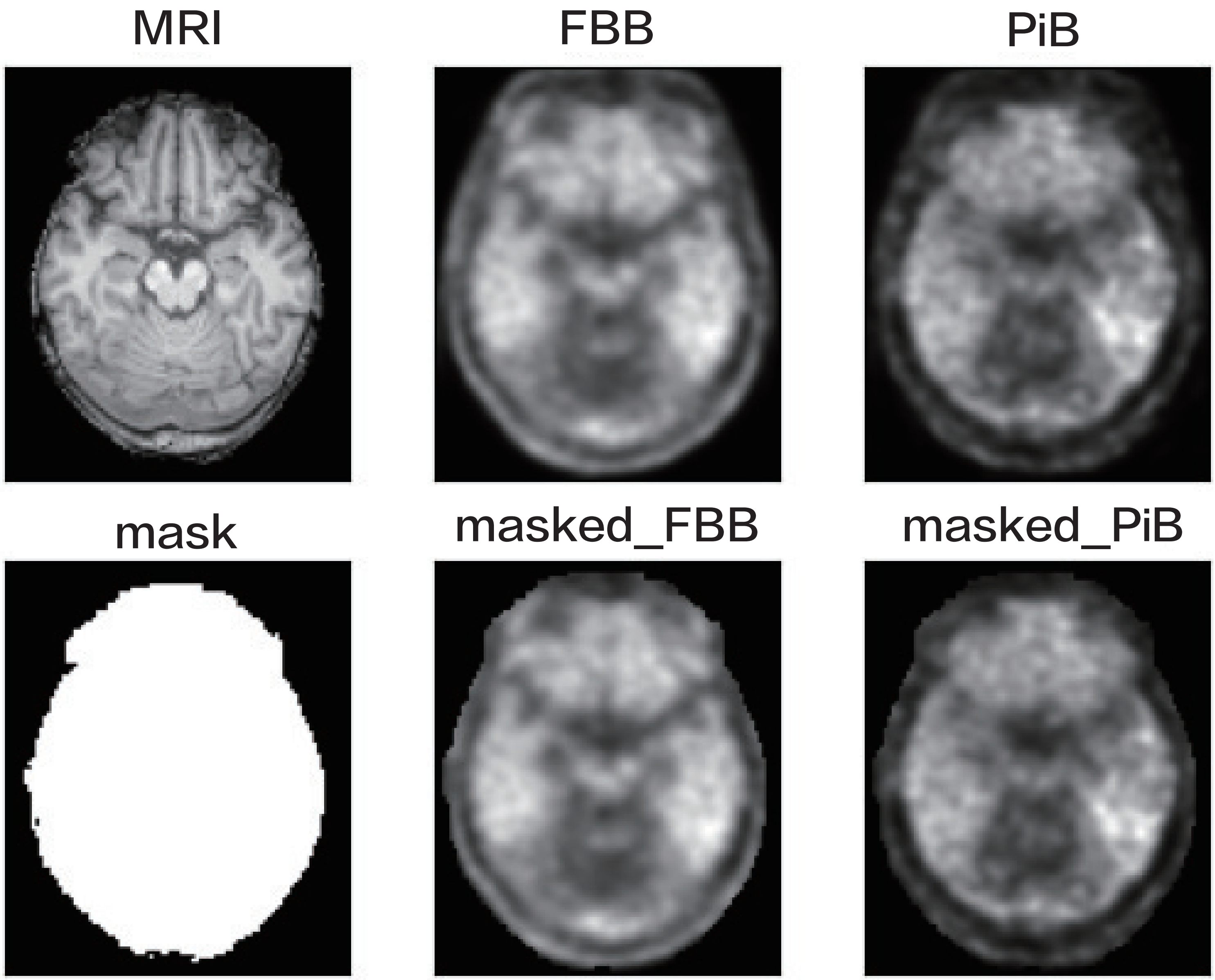

This article is protected by copyright. All rights reserved.
